# Reconstruction of a 3D-printed endoprosthesis after joint-preserving surgery with intraoperative physeal distraction for childhood malignancies of the distal femur

**DOI:** 10.1186/s13018-023-04037-4

**Published:** 2023-07-27

**Authors:** Taojun Gong, Minxun Lu, Li Min, Yi Luo, Chongqi Tu

**Affiliations:** 1 Department of Orthopedics, Orthopaedic Research Institute, West China Hospital, Sichuan University, Chengdu, People’s Republic of China; 2 Model Worker and Craftsman Talent Innovation Workshop of Sichuan Province, No. 37 Guoxue Road, Chengdu, 610041 Sichuan People’s Republic of China

**Keywords:** Joint-preserving, 3D-printed endoprosthesis, Physeal distraction, Femur, Children, Reconstruction

## Abstract

**Background:**

Joint-salvage surgery has been proposed in children with metaphysis malignancy of the distal femur. However, there is still some drawbacks regarding to the surgical technique and endoprosthetic design. In this study, we evaluated the efficacy of a joint-sparing surgical technique for the distal femur in pediatric patients using intraoperative physeal distraction and reconstruction of a 3D-printed endoprosthesis.

**Methods:**

We retrospectively analyzed pediatric patients with distal femoral malignancy who underwent intraoperative physeal distraction and 3D-printed endoprosthetic reconstruction. Clinically, we evaluated functional outcomes using the 1993 version of the Musculoskeletal Tumor Society (MSTS-93) score pre- and post-operation. Complications were also recorded.

**Results:**

Seven children with a median age of 11 years (range 8–15 years) were finally included in our study. The median follow-up time was 30 months (range 27–59 months). The median postoperative functional MSTS-93 score was increased compared with the preoperative scores. The bone-implant interface showed good osseointegration. One patient developed deep infection and another had lung metastasis after surgery. Endoprosthetic complications were not observed.

**Conclusion:**

We recommended that joint-preserving surgery with intraoperative physeal distraction and a 3D-printed endoprosthesis for reconstruction as an option for malignancies of the distal femur in selected pediatric patients.

## Introduction

Pediatric malignant bone tumors predominantly occur in the metaphysis of the distal femur [1, 2]. In previous decades, resection of the epiphysis is necessary to achieve complete tumor excision with negative margins in cases where a malignant tumor had invaded the adjacent metaphysis [3, 4]. Nevertheless, this type of tumor resection, which involves sacrificing the joint, unavoidably compromises the function of the knee joint and the tibial physis, which contributes to around 30% of lower limb growth [1, 4–6].

Considering the significant advancements in multimodal therapies for osteosarcoma and the availability of accurate imaging modalities like magnetic resonance imaging (MRI), joint-sparing resection with preservation of the adjacent joint and ligaments has emerged as an alternative for carefully chosen patients who do not have tumor involvement in the distal femur’s epiphysis [2, 7–9]. This approach ensures improved postoperative function, particularly for young patients with higher functional demands. However, the reconstruction of the femoral diaphysis after joint-preserving surgery presents significant challenges in skeletally immature children [10]. The current available options for reconstruction materials comprise allografts [8], tumor-devitalized autografts [11], vascularized fibular combine with allografts, [10] distraction osteogenesis, [12] and custom-made prosthesis [13]. Despite the advantages of allografts and autografts, such as bone stock preservation and the ability to reattach ligaments or tendons to the graft, they have been associated with a significant incidence of complications, including nonunion, impaired growth, pathological fractures, donor site morbidity, and high infection rates [2, 8]. In addition, surgical complexity, extended periods of immobilization, and weight-bearing restrictions that follow allografts or autografts reconstruction further restrict their clinical applicability [10]. Consequently, a limited number of studies have documented the use of custom-made endoprostheses for reconstructing the intercalary defect [7, 13, 14], which promotes early weight-bearing and functional recovery.

The physis, known for its avascular nature and dense arrangement of chondrocytes, is widely recognized as a natural barrier against malignant invasion [15, 16]. The physis or growth plate, due to the thinning of collagen fibers in this region, is considered the weakest part of the epiphysis, consequently compromising the tensile strength between the epiphysis and metaphysis [16]. When the tumor is situated in the metaphyseal region and does not invade or cross the physis, physeal distraction can be employed to separate the tumor [17]. However, before tumor resection and defect reconstruction, distraction was achieved with the use of an external fixator, allowing for the separation of the epiphysis from the tumor-bearing metaphysis, a process that typically spanned 1–2 weeks. Throughout this period, the procedure should be closely monitored using X-rays, as pediatric patients commonly experience pain [18]. Therefore, surgical techniques should be simplified.

This study aimed to evaluate the efficacy of a joint-sparing surgical technique for the distal femur in pediatric patients using intraoperative physeal distraction. Simultaneously, a customized 3D-printed endoprosthesis was developed for the reconstruction of femoral intercalary defects. A retrospective study was conducted to assess radiographic findings, functional outcomes, oncological results, and associated complications in patients who underwent implantation of 3D-printed endoprosthesis.

## Patients and methods

### Patients involvement

This retrospective study involved the analysis of medical records from pediatric patients who diagnosed with metaphyseal malignancy of the distal femur. The patients underwent joint-sparing surgery and 3D-printed endoprosthetic reconstruction between January 2018 and January 2021. The inclusion criteria were: (1) histologically confirmed diagnosis of malignancy; (2) age of the patients was younger than 16 years; (3) tumor-physis distance ≥ 10 mm with no metastasis before surgery; and (4) follow-up duration exceeding 24 months. Exclusion criteria included patients with incomplete or less than 24 months of follow-up data, poor response to neoadjuvant chemotherapy, or those who underwent alternative reconstruction methods.

Preoperative biopsy was performed on all patients, followed by administration of neoadjuvant and adjuvant chemotherapy. Preoperative radiography and three-dimensional computed tomography (3D-CT) were conducted to assess bone destruction in the affected limb, while MRI was used to evaluate the distance between the tumor and the physis and determine the extent of resection necessary. Bone scans and lung CT scans were conducted to rule out the presence of distant metastases. Statistical analysis was performed using the STATA statistical software (version 16, College Station, TX, USA). A two-tailed *p* value < 0.05 was considered statistically significant.

Our Institutional Review Board approved this study, which adhered to the principles outlined in the Declaration of Helsinki.

### Endoprosthesis design and fabrication

**Fig. 1:**
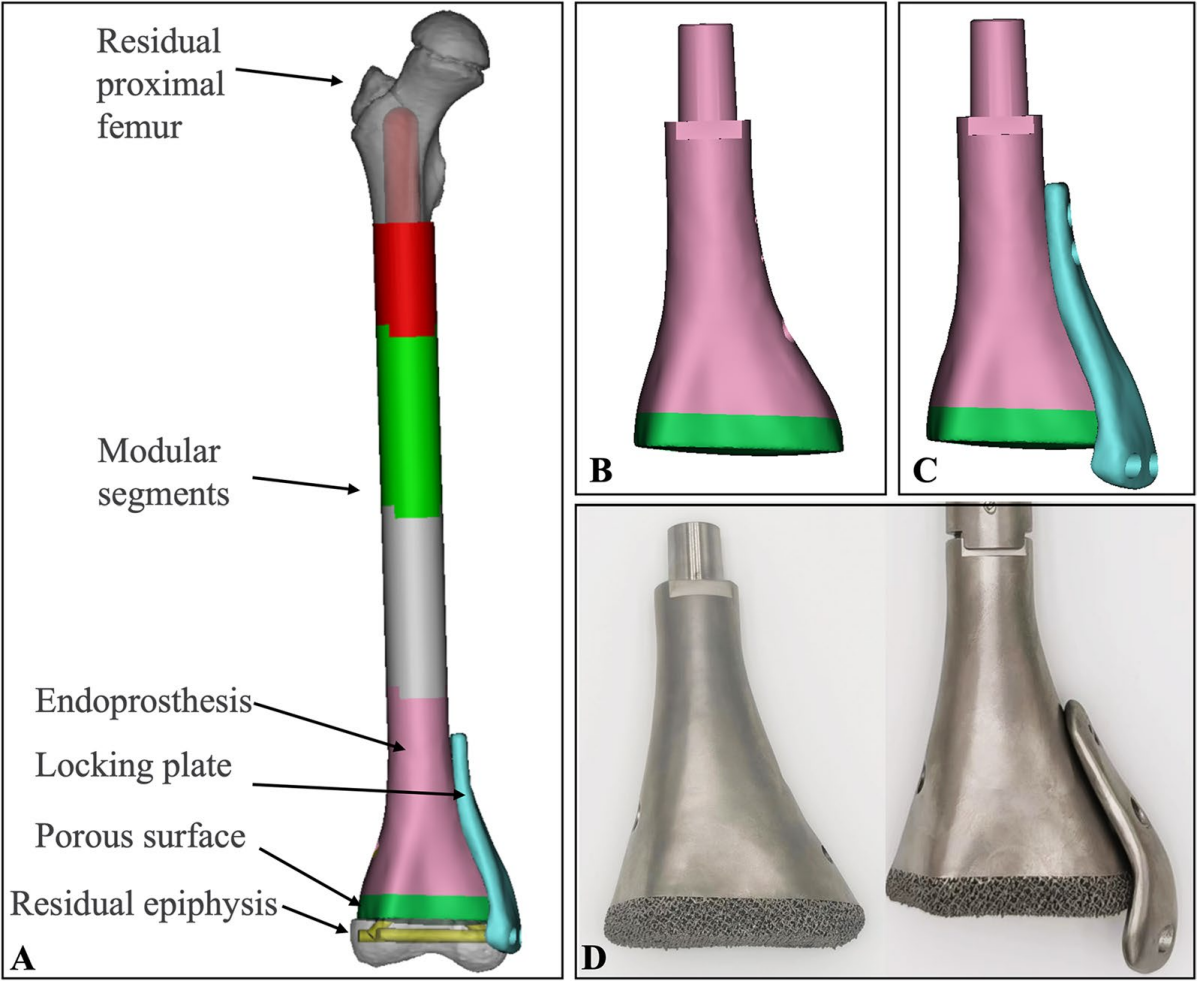
Design and fabrication of the 3D-printed endoprosthesis. **A** Diagram of the endoprosthesis implantation after physeal distraction; **B** and **C** endoprosthesis with solid, porous titanium alloy structures on its distal surface, and a locking plate; **D** the endoprosthesis was fabricated by 3D-printed technology according to the design strategy

Each child’s endoprosthesis was custom-designed by our clinical team and manufactured by Beijing Chunlizhengda Medical Instruments Co., Ltd (Tongzhou, Beijing, China). The initial step involved importing 3D-CT DICOM data into Mimics V20.0 software (Materialise Corp., Leuven, Belgium) to create virtual models of the affected femur. A mirror model of the unaffected contralateral femur served as the prototype for the endoprosthesis. Subsequently, the epiphysis of the prototype was excised to achieve an anatomically consistent interface between the endoprosthesis and the remaining epiphysis following physeal distraction (Fig. 1A). The endoprosthesis was designed to be 60 mm in length and constructed using modular components to accommodate the extent of tumor resection. The endoprosthesis features solid and porous titanium alloy structures on its distal surface that provide adequate mechanical strength and promote bone integration. Subsequently, two to four crossed screw holes were incorporated to fix the endoprosthesis to the femoral condyles (Fig. 1B). Additionally, a separate custom-made locking plate with two transverse screw holes was devised to improve the initial stability of the endoprosthesis (Fig. 1C). Finally, the endoprosthesis was fabricated using the electron beam melting technique (ARCAM Q10plus) with Ti6Al4V alloy (Fig. 1D).

### Surgical technique and postoperative management

**Fig. 2:**
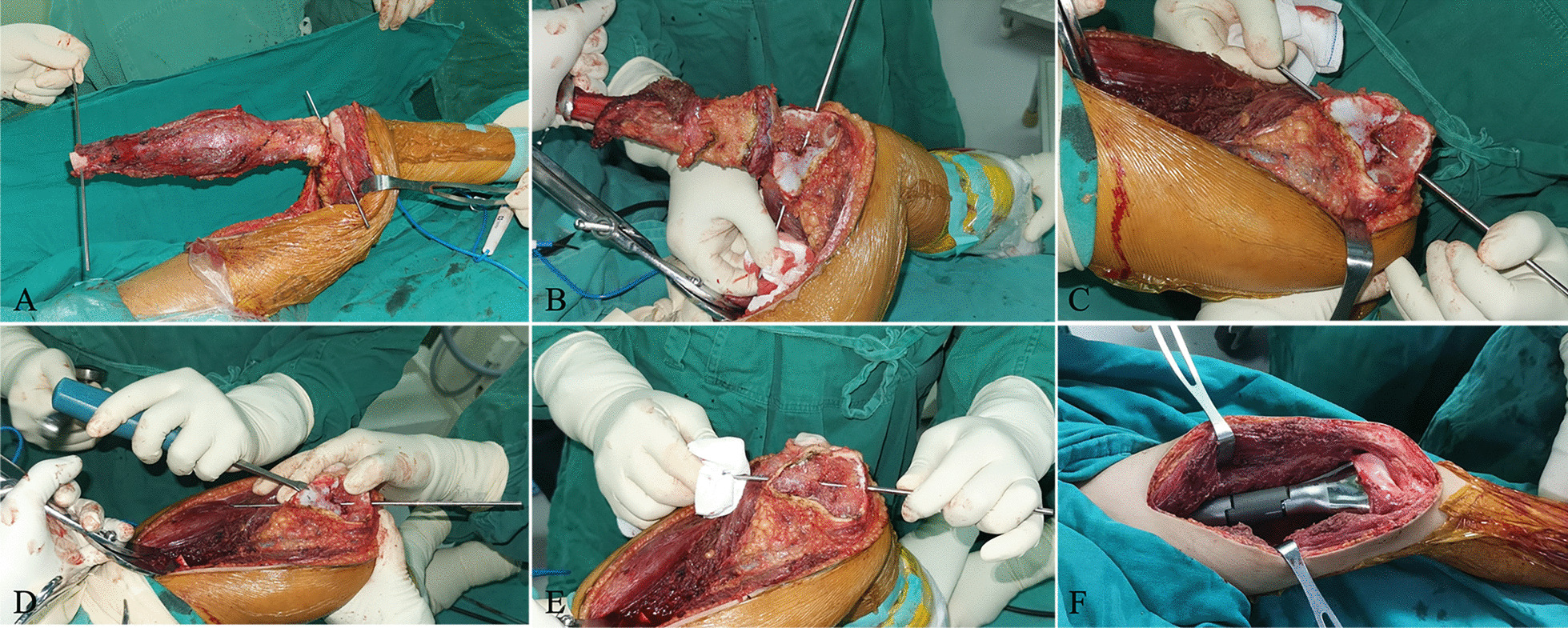
Intraoperative tumor resection and implantation of the 3D-printed endoprosthesis. **A** physeal distraction was performed with the help of two Kirschner wires; **B** the epiphysis was separated from the tumor-bearing metaphysis; **C** open the physis; **D** and **E** removing the surface cartilage of the epiphysis; **F** endoprosthesis implantation with rigid fixation

The same senior surgeon performed all surgeries. Standard surgical approaches were employed for tumor exposure. Proximal femoral osteotomy was conducted, maintaining a safe margin of 3–5 cm from the tumor border based on preoperative MRI findings. The tumor segment was lifted using a bone crocker instrument, and the surrounding soft tissues were dissected. Two Kirschner wires were inserted in parallel—one into the femoral condyle and the other into the remaining stump (Fig. 2A). The border between the epiphysis and physis of the distal femur was identified, followed by longitudinal physeal distraction using the Kirschner wires as anchors (Fig. 2B–C). The knee collateral ligaments and cruciate ligaments were carefully preserved throughout the procedure. Subsequently, the surface cartilage of the epiphysis was removed (Fig. 2D–E), and the endoprosthesis was implanted and adjusted to the desired position. Two to four crossed screws were utilized for endoprosthesis fixation, and a locking plate was added if necessary to enhance initial stability. The proximal femoral canal was reamed to match the diameter of the uncemented stem, which was then implanted using a press-fit technique. The modular segments, ranging from 40 to 120 mm (Chunli Co., Ltd., Tongzhou, Beijing, China), were adjusted to be slightly longer (usually 1 or 2 cm) than the resection length to minimize postoperative leg length discrepancy (Fig. 2F). Finally, a drainage tube was inserted into the incision, and the muscles and soft tissues were meticulously sutured layer by layer.

The knee joint was immobilized with a brace for a period of 2 weeks after the surgery. Functional exercises, including flexion and extension, were initiated 2 weeks after the surgery and followed by a period of 4 weeks with a non-weight-bearing stance. Weightbearing commenced at 8 weeks after the operation and gradually increased in intensity until achieving full weightbearing.

During the follow-up period, all children underwent multiple evaluations, including physical examinations, range of motion (ROM), assessment of clinical symptoms, and imaging examinations of the affected femur and lower extremities. These evaluations were conducted monthly for the first 3 months and then every 3 months. Osseointegration at the surgical sites and leg length were assessed and monitored using radiography and tomosynthesis with Shimadzu metal artefact reduction technology (T-SMART) [19]. Functional outcomes were evaluated using the Musculoskeletal Tumor Society (MSTS-93) score, based on the 1993 version. This score ranges from 0 to 30, with a higher score indicating better functional outcomes. [20] Related complications including infection, wound healing problem, periprosthetic fracture, local recurrence, leg length discrepancy, and metastasis were recorded at latest follow-up.

## Results

This retrospective study analyzed seven pediatric patients with femoral osteosarcoma, consisting of four boys and three girls, with a median age of 11 years (range 8–15 years) at the time of surgery. The median follow-up time was 30 months (range 27–59 months). Five patients were diagnosed with conventional osteosarcoma, one patient with periosteal osteosarcoma, and one patient with telangiectatic osteosarcoma. All patients were classified as having stage IIB disease according to the Enneking staging system. [21] The median ROM of the knee was 120° (range 90°–130°). The median functional MSTS-93 score improved from 17 (range 12–20) preoperatively to 28 (range 24–30) postoperatively (*p* < 0.001). The median resection length was 235 mm (range 128–312 mm). The median distance between tumor and physis was 17 mm (range 13–25 mm). The median leg length discrepancy was 11 mm (range 0–22 mm). All patients reached a surgical margin of R0 confirmed by postoperative pathological results. Patient demographics are summarized in Table 1.

**Table 1:** Demographics of the 7 patients underwent physeal distraction and 3D-printed endoprosthesis reconstruction of distal femur

Patient	Age (years)	Gender	Diagnosis	Side	Enneking staging	DBTP (mm)	RS (mm)	LLD (mm)	Follow-up time (month)	Chemotherapy circles		MSTS-93		Complication
										Pre-op	Post-op	Pre-op	Post-op	
1	11	F	COS	Left	IIB	19	142	22	59	3	6	15	28	NA
2	14	M	COS	Right	IIB	25	277	6	49	3	6	13	25	Deep infection
3	11	F	COS	Right	IIB	13	163	11	30	3	6	12	28	NA
4	9	M	TOS	Right	IIB	15	128	0	29	3	7	20	29	NA
5	8	M	COS	Right	IIB	17	259	20	27	2	7	17	29	Pulmonary metastasis
6	13	F	POS	Right	IIB	17	235	13	38	3	5	18	27	NA
7	15	M	COS	Right	IIB	20	312	0	27	3	7	19	30	NA
Median	11					17	235	11	28		17	27		

*F* female, *M* male, *DBTP* distance between tumor and physis, *RS* resection length, *LLD* leg length discrepancy, *Pre-op* pre-operation, *Post-op* post-operation, *COS* conventional osteosarcoma, *TOS* telangiectatic osteosarcoma, *POS* periosteal osteosarcoma, *NA* not applicable, *MSTS* Musculoskeletal Tumor Society

**Fig. 3:**
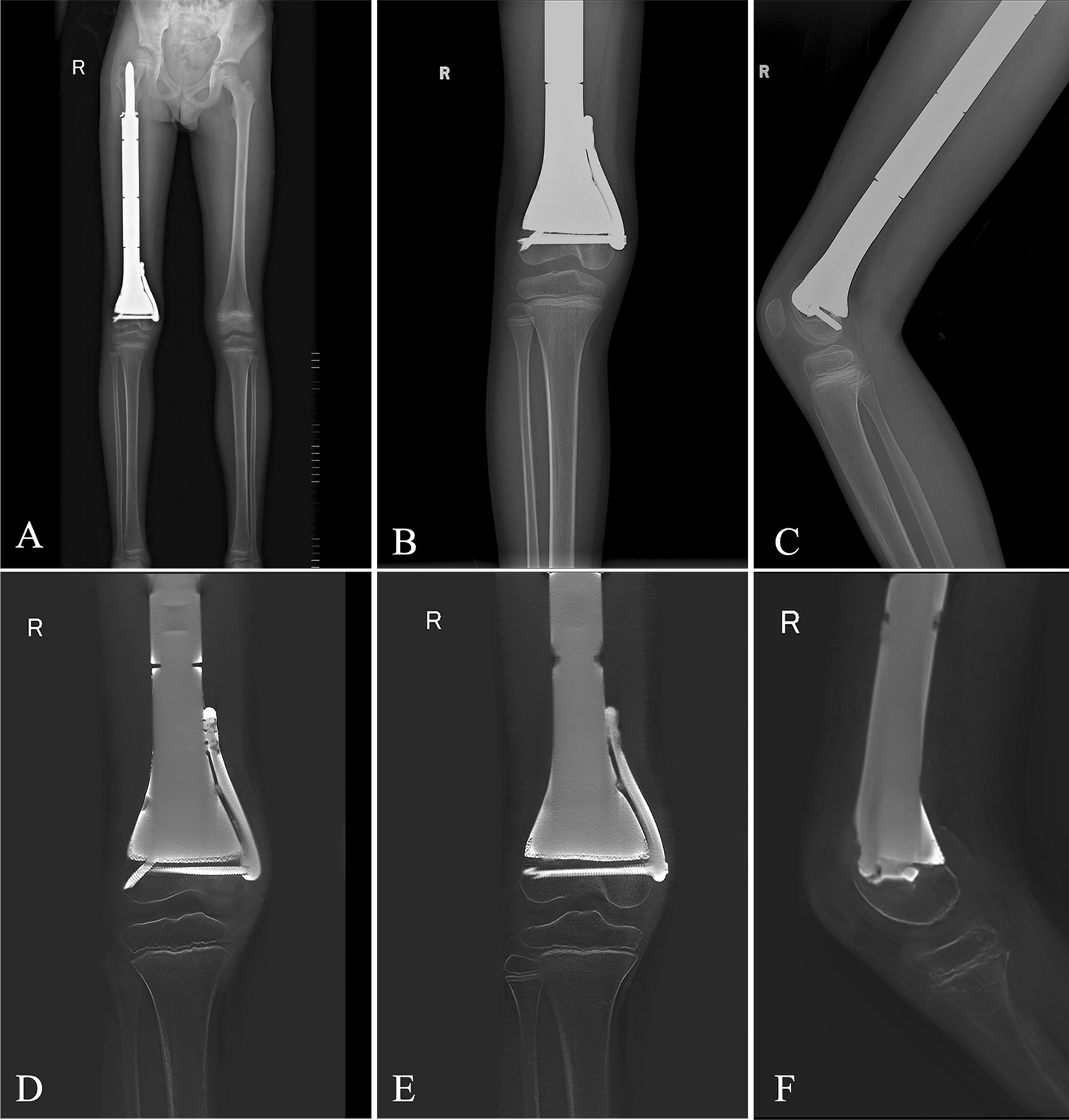
Postoperative radiographs. **A**–**C** plain radiograph shows the proper position of the 3D-printed endoprosthesis; **D**–**F** digital tomosynthesis image shows good osseointegration at the interface between the host bone and the porous surface

No endoprosthetic complications, such as displacement, breakage, or periprosthetic fracture, were observed in terms of radiographic outcomes and complications (Fig. 3A–C). The bone-implant interface showed good osseointegration, as demonstrated by T-SMART imaging (Fig. 3D–F). One patient (patient 2) experienced deep infection, resulting in the removal of the endoprosthesis. An isolated pulmonary metastasis was detected in another patient (patient 5) 16 months after the operation and received chemotherapy combined with targeted therapy. All patients were alive at the last follow-up. (Table 1).

## Discussion

Primary bone malignancies in children most commonly occur in the metaphysis of the distal femur, representing approximately 35% of all skeletal malignant tumors [22, 23]. Generally, limb-sparing surgery, involving complete excision of the knee joint and subsequent replacement with an extendable or non-extendable modular endoprosthesis, was the predominant approach [4, 6, 24]. However, this reconstructive method was associated with long-term complications and posed the risk of distal femoral epiphysis loss and disruption of the normal physis of the proximal tibia in skeletally immature patients [3, 6]. Therefore, certain researchers have advocated for the utilization of joint preservation surgery in specific cases to retain the knee joint and safeguard the growth of the remaining epiphysis [2, 7, 9, 25]. Accurate preoperative imaging, particularly MRI, plays a crucial role in identifying pediatric patients suitable for extensive tumor resection and joint-salvage surgery. According to San-Julian and colleagues, the invasion of the metaphysis by sarcoma in children is categorized into three types: type I, where the distance between the lesion and the epiphysis is greater than 2 cm; type II, where the distance between the lesion and the epiphysis is less than 2 cm or they are adjacent; and type III, where the lesion has partially infiltrated the epiphysis [25]. Furthermore, the physis, also known as the growth plate, was considered to act as a barrier, impeding tumor spread [11]. Consequently, joint-sparing surgery was recommended when the tumor was at least 10 mm away from the physis [2, 11, 16]. This study determined that the median distance between the tumor and the physis was 17 mm (range 13–25 mm), as confirmed by preoperative MRI scans. Consequently, joint salvage surgery was deemed suitable for all pediatric patients included in this study.

Numerous researchers have documented various reconstructive materials and surgical techniques utilized in joint-preservation surgery for children.[7, 8, 10, 11, 13, 18, 26] (Table 2) Presently, the available options comprise biological reconstruction and prosthetic reconstruction. Biological materials encompass allografts, tumor-devitalized autografts, and vascularized fibular grafts [2]. Aponte-Tinao and colleagues retrospectively analyzed 35 pediatric patients (mean age 9 years) who underwent tumor resection with epiphysis preservation and subsequent reconstruction utilizing intercalary allografts. The study population exhibited an overall survival rate of 86% at both the 5-year and 10-year marks, while the mean functional MSTS score at final follow-up was 26 points (range 10–30 points). Nevertheless, a significant proportion of complications related to allografts occurred in 16 patients, resulting in the removal of 10 allografts. These complications included 2 infections, 11 fractures, and 3 nonunion. [8] In order to mitigate the incidence of complications associated with the avascular nature of massive bone allografts, the combination of allografts and vascularized free fibulas has been recommended [27]. Errani et al. conducted a comparison of two reconstruction techniques: one utilizing a massive bone allograft alone and the other incorporating a vascularized free fibula, in children who underwent intercalary resection of femoral bone sarcomas. Their findings revealed no significant difference in the survival of reconstructions between the two methods. They concluded that a vascularized fibula should only be employed to salvage the allograft in cases of fracture or nonunion [10]. However, the utilization of vascularized free fibulas was associated with complex surgical techniques, a risk of donor site morbidity, and a lengthened rehabilitation process. Partial weightbearing was permitted at an average of 2–4 months after the operation, while full weightbearing was deferred until an average of 8–10 months [10, 28].

**Table 2:** Review of previous studies on joint-persevering surgery in children

Study	Number of patients	Diagnosis	Reconstruction method	Follow-up time	Complications	Functional outcomes
Wong et al. [7]	8 (5 PPs^a^)	CS, OS	Custom prosthesis and autografts	Mean 41 months; (range 25–60 months)	Lung metastasis, aseptic loosening	Mean MSTS score 29.1, mean knee flexion 115°
Aponte-Tinao et al. [8]	35 (20 PPs)	OS	Allograft	Mean 9 years (range 1 and 16 years)	Fracture, infection, nonunion, LR	Mean MSTS score 26
Errani et al. [9]	46 (46 PPs)	OS, ES	Allograft or with a ascularized fibula	Median 123 months (range 28–261 months)	Fracture, infection, nonunion, lung metastasis	MSTS score 25.9 ± 3.5 and 26.7 ± 2.3, respectively
Takeuchi et al. [10]	12 (12 PPs)	OS	Tumor-devitalized autograft	Mean 63 months (range 41 and 90 months)	Fracture, infection, LR, LLD	Mean MSTS score 27.7
Liu et al. [12]	12 (3 PPs)	OS, fibrosarcoma	3D-printed prosthesis	Mean 22.5 months (range 7 and 32 months)	Infection, LR, lung metastasis	Mean MSTS score 28
Betz et al. [17]	6 (6 PPs)	OS, ES	Physeal distraction, allograft or vascularized fibula	Median 62 months (range 18–136 months)	Delayed wound healing, infection, nonunion, LLD	Mean MSTS score 79%, mean TESS 83%
Gao et al. [25]	10 (10 PPs)	OS	Physeal distraction, allograft	Mean 38.5 months (range 15 and 56 months)	Lung metastasis, infection,	Mean International Society of Limb Salvage score 21.8
Presented study	7 (7 PPs)	OS	Intraoperative physeal distraction, 3D-printed modular prosthesis	Median 30 months (range 27–59 months)	Lung metastasis, infection	Median MSTS-93 score 27

*PPs* pediatric patients, *CS* chondrosarcoma, *ES* Ewing’s sarcoma, *OS* osteosarcoma, *MSTS* Musculoskeletal Tumor Society, *LR* local recurrence, *TESS* Toronto Extremity Salvage Score, *LLD* limb length discrepancy

^a^The pediatric patients are defined as those under 16 years of age

Prosthetic reconstruction has gradually been attempted for repairing intercalary defects of the femur, as it enables early weight-bearing and promotes functional recovery [7, 13]. Wong et al. reviewed six cases, which involved five pediatric patients undergoing joint-sparing tumor resection utilizing image-guided computer navigation and reconstruction with a custom-made prosthesis and autografts. The patients were initially permitted to walk with a brace and engage in partial weight bearing for 4 weeks, after which they were allowed to bear full weight. The functional outcomes were favorable, with a mean MSTS score of 29.1 (range 28–30) and knee flexion of up to 130° [7]. In our study, weight bearing was initiated 4 weeks after surgery, and the median functional MSTS-93 score was 28 (range 24–30), which is consistent with previous studies.

In another retrospective study, Liu et al. developed an osteotomy guide plate for joint-preserving intercalary tumor resection and reconstruction in adults using a 3D-printed endoprosthesis. The study reported accurate fitting between the residual bone and the prosthesis, resulting in good postoperative function [13]. However, tumor resection assisted by an osteotomy guide plate will inevitably lead to the sacrifice of bone stock in the residual epiphysis in children due to its irregular shape. As an alternative for resecting pediatric bone sarcomas, physeal distraction can be utilized to mitigate this issue [17, 18, 26]. This technique, initially reported by Canadell et al. in 1994, enables the separation of the epiphysis from the tumor-bearing metaphysis [17]. Betz and colleagues conducted a study involving 6 children with primary metaphyseal malignancy who underwent physeal distraction as part of tumor resection, followed by reconstruction using massive bone allograft, autograft, or a combination of both. No local recurrence was observed in any of the patients, and the mean MSTS score at the last follow-up was 79% (range 53–97%). Whereas, the procedure was complicated. Prior to tumor resection and reconstruction, an external fixator was placed to separate the epiphysis from the tumor-bearing metaphysis. Meanwhile, physis rupture occurred unpredictably and often caused discomfort. It also increased the risk of pin tract infection [18].

In order to simplify the surgery and minimize the discomfort caused by the external fixator, physeal distraction was performed during the operation using two parallel Kirschner wires in our study. This method was chosen due to the weak tensile strength at the metaphysis-epiphysis junction [16, 17], which was prone to separate under artificial force. Subsequently, the defect was reconstructed using a 3D-printed endoprosthesis. Meanwhile, primary stability was enhanced by crossed screws and a separate custom-made locking plate. All children exhibited satisfactory functional outcomes, and there were no instances of local recurrence during the latest follow-up assessment. The median leg length discrepancy was 11 mm (range 0–22 mm), a measurement considered acceptable for children.

We acknowledge that our study has several limitations. Firstly, the limited sample size of only seven patients is a notable limitation. The heterogeneity within a small patient population may have further affected the accuracy of the results. Additionally, the lack of a comparative cohort study prevents the determination of the advantages of the surgical technique and the endoprosthetic design. Finally, the long-term outcome of the 3D-printed endoprosthesis remains unclear, necessitating further investigation.

## Conclusions

In this study, we observed favorable functional results by employing joint-preserving surgery with intraoperative physeal distraction and a 3D-printed endoprosthesis to reconstruct the intercalary defect of the distal femur in pediatric patients. We recommend considering intraoperative physeal distraction combined with a 3D-printed endoprosthesis as a viable alternative for selected pediatric patients diagnosed with metaphyseal malignancy of the distal femur.

## Data Availability

The datasets used and analyzed during the current study are available from the corresponding author on reasonable request.
